# The Potential Impact of Density Dependent Fecundity on the Use of the Faecal Egg Count Reduction Test for Detecting Drug Resistance in Human Hookworms

**DOI:** 10.1371/journal.pntd.0000297

**Published:** 2008-10-01

**Authors:** Andrew C. Kotze, Steven R. Kopp

**Affiliations:** 1 CSIRO Livestock Industries, St. Lucia, Brisbane, Queensland, Australia; 2 School of Veterinary Science, University of Queensland, Brisbane, Queensland, Australia; George Washington University, United States of America

## Abstract

Current efforts to control human soil-transmitted helminth (STH) infections involve the periodic mass treatment of people, particularly children, in all endemic areas, using benzimidazole and imidothiazole drugs. Given the fact that high levels of resistance have developed to these same drugs in roundworms of livestock, there is a need to monitor drug efficacy in human STHs. The faecal egg count reduction test (FECRT), in which faecal egg output is measured pre- and post-drug treatment, is presently under examination by WHO as a means of detecting the emergence of resistance. We have examined the potential impact of density dependent fecundity on FECRT data. Recent evidence with the canine hookworm indicates that the density dependent egg production phenomenon shows dynamic properties in response to drug treatment. This will impact on measurements of drug efficacy, and hence drug resistance. It is likely that the female worms that survive a FECRT drug treatment in some human cases will respond to the relaxation of density dependent constraints on egg production by increasing their egg output significantly compared to their pre-treatment levels. These cases will therefore underestimate drug efficacy in the FECRT. The degree of underestimation will depend on the ability of the worms within particular hosts to increase their egg output, which will in turn depend on the extent to which their egg output is constrained prior to the drug treatment. As worms within different human cases will likely be present at quite different densities prior to a proposed FECRT, there is potential for the effects of this phenomenon on drug efficacy measurements to vary considerably within any group of potential FECRT candidates. Measurement of relative drug efficacy may be improved by attempting to ensure a consistent degree of underestimation in groups of people involved in separate FECRTs. This may be partly achieved by omission of cases with the heaviest infections from a FECRT, as these cases may have the greatest potential to increase their egg output upon removal of density dependent constraints. The potential impact of worm reproductive biology on the utility of the FECRT as a resistance detection tool highlights the need to develop new drug resistance monitoring methods which examine either direct drug effects on isolated worms with in vitro phenotypic assays, or changes in worm genotypes.

## Author Summary

Current efforts to control soil-transmitted helminth (STH) infections in humans in endemic countries involve the mass administration of drugs. The use of these same drugs for many years to control livestock nematodes has resulted in the emergence of significant levels of resistance. Hence, there is a need to manage the use of drugs against human STHs in order to reduce the likelihood of resistance developing. An important component of managing drug use will be an ability to detect drug resistance should it emerge. WHO and the World Bank are presently supporting initiatives to develop tools for detecting drug resistance in human STHs. The tool to be assessed in the short term is the faecal egg count reduction test (FECRT). We have examined literature on an aspect of worm reproductive biology with potential to impact significantly on the FECRT. We describe the potential effects that density dependent egg production by female hookworms may have on interpretation of FECRT data. This study highlights a potential weakness in reliance on the FECRT for assessment of drug resistance in human hookworms, hence emphasising the need to develop more advanced worm bioassay and molecular methods.

## Introduction

Soil transmitted helminth (STH) infections (*Ascaris lumbricoides*, *Trichuris trichiura,* and the hookworms *Necator americanus* and *Ancylostoma duodenale)* contribute significantly to morbidity in humans in endemic countries. The major means of controlling these infections is by the periodic administration of anthelmintic drugs [1]. Given the lessons from livestock, where resistance to anthelmintics is widespread [2], the potential for anthelmintic resistance in human parasites has been recognized [3]. There are a number of differences in drug use patterns between the human and livestock fields which may aid in reducing the rate of resistance development in the former [4],[5], however, the potential for resistance in human parasites is a significant issue [6].

It is clear that monitoring of drug resistance will be an important part of ensuring the success of current efforts to control STH infections. Although sensitive molecular tests are able to detect SNPs associated with benzimidazole resistance in livestock nematodes, they are still not available for human STHs. Phenotypic assays of worm eggs or larvae *in vitro* are useful for detection of resistance to some drug groups in livestock nematodes, however, their use with human STHs requires substantial validation and field testing. Hence, the faecal egg count reduction test (FECRT), in which egg output per gram of faeces (epg) is compared in individuals before and after drug treatment to estimate drug efficacy, is considered to be the most useful technique currently available for detecting the emergence of resistance in STH infections in humans.

A recent study by Kopp et al. [7], in which dogs infected with the hookworm *Ancylostoma caninum* were treated with pyrantel, showed a poor relationship between drug efficacy and changes to epg. A mean drug efficacy of 71% in two dogs infected with an isolate showing low level resistance to the drug was associated with a 41% increase in epg. That is, egg output increased markedly despite the removal of a significant proportion of the adult worm burden by the drug. They suggested that this was due to an increase in egg production in surviving adult female worms after drug treatment due to the relaxation of density-dependent constraints on egg production.

Density dependent fecundity has been demonstrated many times with Helminth parasites [reviewed by 8],[9]. The effect is seen as a decrease in egg output as the parasite burden increases. An additional feature typical of reports on density dependent fecundity is the presence of a greater degree of variation in fecundity at low worm burdens. However, the phenomenon is not associated with all Helminth infections, for example, several reports have indicated an absence of density dependent effects on the fecundity of *Haemonchus contortus* in sheep [10],[11], while the evidence for *Ostertagia circumcincta* is equivocal [12], and Shaw and Moss [13] found no evidence of a density dependent effect on fecundity in *Trichostrongylus tenuis* in red grouse.

In terms of human STHs, the density dependent effects have been reported for *Ascaris lumbricoides* [14]–[17], *Necator americanus* [18],[19], and mixed *N. americanus* and *Ancylostoma duodenale* infections [20]. Anderson and Schad [20] noted that while the intrinsic fecundities of the two human hookworm species were similar, the density dependent constraints on egg production were more severe for *N. americanus*. For *Trichuria trichiura* the evidence is less clear. Bundy et al. [21] reported a strong density dependent effect on fecundity, while a later study [22] found that no significant association between fecundity and worm burden. The later report did however note that very high per capita fecundities were only observed at low worm burdens. Michael and Bundy [23] reported density dependent effects on fecundity in *Trichuris muris*. Density dependent effects on egg output have also been reported for the canine hookworm *A. caninum* [24],[25].

It has been suggested that density dependent fecundity results from either competition for resources between parasites, or from immunological responses by the host [8],[9], or from direct parasite-parasite interactions [26]. In the case of *Strongyloides ratti* infections in rats, the density dependent effects on the parasite's establishment, survivorship, as well as fecundity, are mediated by host immune responses [27],[28].

A number of reports have described the impact of density dependent fecundity on transmission dynamics [20],[29], the rate of parasite reinfection following chemotherapy [30], and the spread of drug resistance [31]. We were interested in examining the effect that the phenomenon may have on the use of the FECRT to detect anthelmintic resistance in human STHs. If egg-laying by female hookworms is constrained by density dependent effects, the question arises as to whether this phenomenon is dynamic in nature if the worm density within a host is changed by, for example, a drug treatment. Survivors of a drug treatment will be present at a lower density after the treatment compared to before. Will the constraints on egg production be removed as the worm density decreases? The present study aimed to address this issue in terms of its potential to distort a FECRT by examining recent evidence on the response of the canine hookworm to drug treatment [7], and by examining an early *N. americanus* dataset describing the relationship between egg output and worm infection levels [18].

## Methods

### Analysis of canine hookworm data

Kopp et al. [7] examined the response of canine hookworms to pyrantel drug treatment. The hookworms were from a highly resistant isolate, and an isolate showing a low level of resistance. Infections were established in four dogs (two per worm isolate), and faecal egg counts were measured daily. Egg counts stabilised by day 24, and all dogs were treated with pyrantel. Egg counts were monitored for a further 6 days, and faeces was collected daily. The dogs were then euthanased and all worms present in the small intestine were counted. Total pretreatment worm counts were calculated as the sum of those present in the animal after drug treatment and those recovered from the daily feacal collections. Egg output (epg) data over the time course of the experiment was reported. For the present study we reanalysed the data from Kopp et al. [7] in terms of egg output per day (epday) per worm, rather than epg. Daily faecal weights had not been recorded so in order to do estimate daily egg output per dog we used a mean value for faeces output of dogs of similar size to those used in the Kopp et al. [7] study that were housed under identical conditions for an earlier experiment. This represents an approximation of the faeces output for each of the 4 study dogs; however the absolute amount of output is not crucial to our analysis, as long as it remained approximately constant before and after drug treatment. Kopp (pers. comm.) noted that there was no observable change in faecal output in each dog post treatment.

### Analysis of human hookworm data

We examined the data of Hill [18] who reported egg output (epg, and epday per female) and worm burden (counting worms in faeces after treatment with carbon tetrachloride and oil of chenpodium) in human cases infected with *N. americanus* in Porto Rico in 1926. Complete data was obtained for 93 cases. Hill [18] noted a threshold of 500 females above which egg production per female was always low. We grouped the human cases separately: those above the 500 threshold which we term as ‘constrained’ in their egg output by density effects (n = 11), and the remainder of the cases with less than 500 worms (n = 82). We examined the effects on a FECRT if these ‘constrained’ worms are able to increase their egg output upon relaxation of the density dependent constraints by a drug treatment. We assumed that as a result of the removal of significant proportions of the adult worm burden (60–90%), all female worms in the ‘constrained’ group would be able to increase their egg output to the median value of those worms lying below the 500 female threshold. This represents an estimation of the post drug egg output as the worms in the 11 cases will most likely adopt a range of egg outputs after drug treatment as reflected by the great degree of variability shown by worms at low densities [8],[9]. We assumed that all worms below the 500 threshold (n = 82) would not change their egg output per female after drug treatment, as the drug treatment did not represent the removal of fecundity constraints. We calculated the numbers of female worms remaining in all 93 human cases at each drug efficacy value, and then expressed their egg output on the basis of the faecal output of each human case prior to the drug treatment (mean of 2 or 3 faecal output values given for each human by Hill [18]). The % decrease in faecal egg count (% FECR) for each person was then calculated using the formula: [(T1-T2)/T1]×100, where: T1 = epg before treatment,T2 = epg after treatment. Mean % FECR was calculated for the constrained (n = 11) and unconstrained (n = 82) groups, as well as for the whole population (n = 93).

We also examined the relationship between epg and infection level for the Hill [18] data set. The human cases were subdivided into those showing epg values above or below 10,000, 5,000 and 2,500. The relationship between these groupings and actual infection levels (number of female worms per host) was then examined graphically. This allowed assessment of the impact of removing certain epg groups from the full data set on the ability to preferentially omit high infection level cases from a FECRT analysis.

## Results

**Figure 1:**
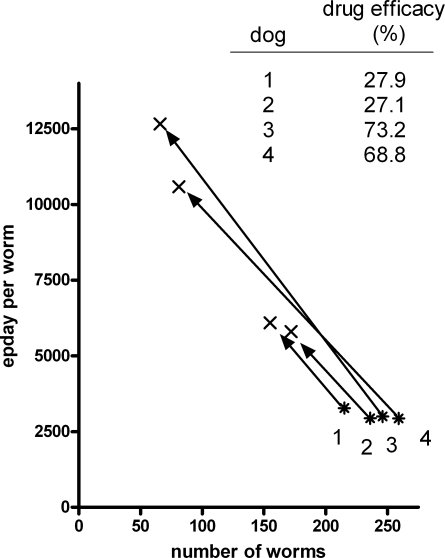
Increased egg output by canine hookworms surviving a drug treatment. Analysis of the data of Kopp et al. [7] to indicate changes in egg production by *A. caninum* worms surviving a treatment of their host animals with pyrantel. Dogs 1 and 2 were infected with an isolate of high level resistance to the drug, while dogs 3 and 4 were infected with an isolate showing low level resistance; * = prior to drug treatment, x = 6 days after treatment.

Figure 1 shows an analysis of the egg output and adult worm numbers for the 4 dogs before and after treatment with pyrantel using the data of Kopp et al. [7]. Dogs 1 and 2 were infected with worms from an isolate with a high level of resistance to the drug, while dogs 3 and 4 were infected with an isolate which showed only a low level of resistance. All four dogs had similar worm burdens before the drug treatment, and all showed very similar rates of egg production per adult worm. Following drug treatment, all dogs showed an increase in their egg output per worm alongside reductions in worm numbers. The change in worm reproductive output was most dramatic for dogs 3 and 4 which increased egg output per worm by 4.2- and 3.6-fold, respectively.

**Figure 2:**
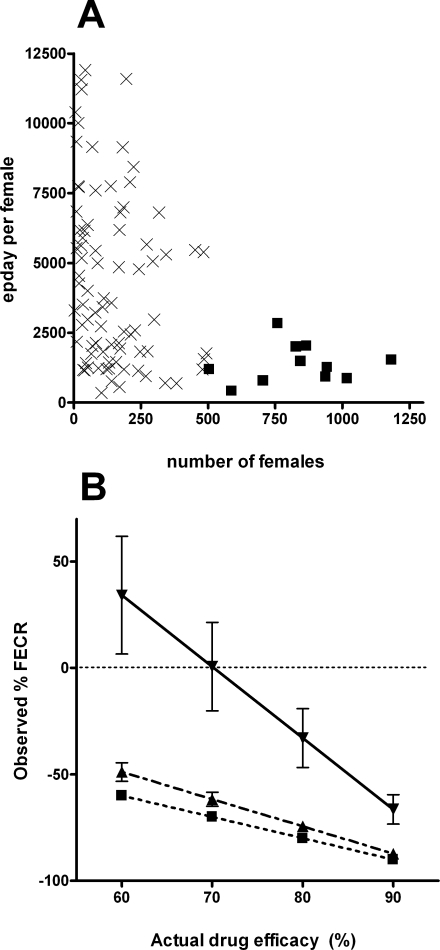
Examination of *N. americanus* egg output data from Hill [18]. A = egg output per female worm for the 93 human cases; x  = cases with <500 females (n = 82); ▪ = cases with >500 females (n = 11) (see text for details). B: Relationship between % FECR in response to treatment with a drug of known efficacy for the human cases in the < or > 500 female groups; ▪ dotted line: cases with pretreatment female numbers <500 (assuming no increase in egg output per female after treatment) (n = 82); ▾solid line: cases with pretreatment female numbers >500 (assuming post treatment egg output per female equals the median of the <500 group) (n = 11); ○ dashed line: all cases combined (n = 93). % FECR is based on a simple model in which the worms surviving the drug treatment in the >500 group from A are able to increase their egg output to match the median output of the <500 group from A, while the <500 output group show no change in output following drug treatment. Each data point represents mean±SE (n = 82, 11 or 93) (see text for details).

The production of eggs per day per female worm from the study of Hill [18] is shown in Figure 2A. The two trends noted earlier as common to such egg output data are apparent, namely, 1) as the number of worms harboured increases there is a decrease in the number of eggs produced per female, with no cases of high egg production per female occurring at high worm densities (>500 females per person), and 2) at lower densities (<500 females per person) there is a large degree of variation in egg production between females. Egg production per day per female at densities less than 500 females varied from 351 to 11,904.

The result of our simulated FECRT on the human cases described in the Hill [18] data set is shown in Figure 2B. The actual drug efficacy was varied from 60–90%, while observed efficacy was calculated by assuming that the ‘constrained’ cases (those shown as squares in Figure 2A) could increase their egg output to the median of the unconstrained cases (indicated by crosses) due to the relaxation of density dependent constraints. It is apparent that a grouping of the 11 highest infection level human cases shows a % FECR significantly less than the actual removal of worms by the drug, and that this component of the population has affected the ability of the whole data set (93 cases) to accurately reflect drug efficacy.

**Figure 3:**
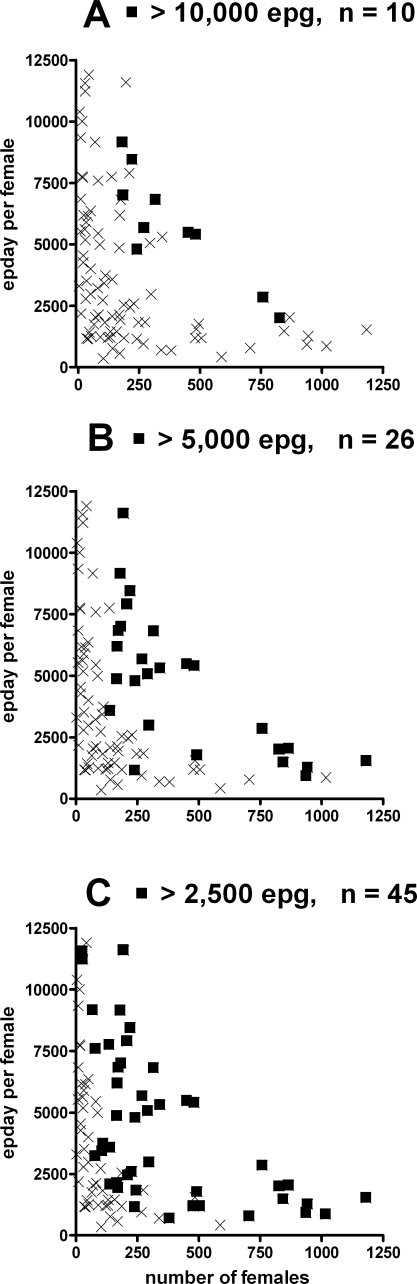
An examination of epg prior to drug treatment as a predictor of the level of density dependent fecundity constraint imposed on worm populations using the data set of Hill [18]. Parts A, B, C highlight cases with epg >10,000, >5,000, or >2,500, respectively, as ▪; all other cases are shown as x.

Figure 3 highlights the cases from Hill [18] which fall into various epg groupings in order to demonstrate the effect of using such pre-treatment epg values to select appropriate candidates for a FECRT. It is apparent that the highest epg cases (epg >10,000, n = 10) do not coincide with the heaviest infection levels (Figure 3A). As the epg cut-off decreases, the highest infection level cases are mostly highlighted, alongside many cases with much lower infection levels (Figures 3B, 3C). For this data set, removal of most of the heaviest infections (ie. >500 females) from a FECRT would require the omission of all cases with epg >2,500, representing the additional omission of many low infection (<500 female) cases. It is clear that the relationship between egg output for each human case (ie, epg) and infection level (number of females) is poor due to the extent to which egg output per female can increase at low worm densities.

## Discussion

The study by Kopp et al. [7] suggests that the reproductive behaviour of adult hookworms before and after a drug treatment will likely be far from static. The significant increase in egg production by the worms following drug treatment is most likely a consequence of the relaxation of density dependent constraints on their egg output. The degree to which egg output increased coincided with the extent to which the worm populations were reduced by the drug treatment in the dogs harbouring the two worm isolates showing different levels of resistance to the drug. Given the widespread occurrence of density dependent worm fecundity constraints in human STHs [14]–[22] it is most likely that the phenomenon noted here with the canine hookworm will apply more widely to the human STHs.

The effect of the host immune response on fecundity in *S. ratti* has been demonstrated to be reversible [32],[33], with the immune mediated constraints on egg production being quickly reversed upon removal or suppression of the host immune effects. The time scale of the reversibility in these studies is similar to that in the report of Kopp et al. [7] (6 days), and as would occur in a FECRT (approximately 10 days between drug treatment and faecal egg count). However, the role of host immune mechanisms in the increased fecundity observed by Kopp et al. [7] is unclear as no specific suppression or removal of host immunity was imposed as in the experiments described above for *S. ratti*.

It is likely that among a group of people involved in a FECRT will be some cases in which female worms are producing more eggs post-treatment than they did prior to the drug treatment due to the relaxation of density constraints following the drug treatment. Therefore epg in these cases would be expected to decrease by a lower percentage than worm burden in response to the drug. Hence, a comparison of epg before and after drug treatment will underestimate efficacy. For dogs 3 and 4 from Kopp et al. [7] removal of 71% of the worm burden by the drug was associated with an increase in epg of 41%. The degree to which an increase in fecundity can distort FECRT data will depend on the extent to which the egg production of female worms is constrained prior to the FECRT. It is clear that while worms which are constrained in their egg output by density-dependent effects prior to the treatment will have the potential for increasing their egg output per worm as worm numbers decrease, cases whose worms are at low densities and hence may be producing eggs at nearer to their reproductive potential within that host prior to the drug, will have a significantly lower scope to further increase egg output. It can be seen from Figure 2B that the overall FECR data from a pool of cases will depend on the relative proportions of worms that are able to increase egg output significantly in response to drug treatment, compared to those which do show little or no change, and the extent of any output increases in individual cases. For FECRT data to be a useful tool for detecting changes in drug efficacy (that is, drug resistance), it is important for such population based variables to be minimised. If FECRTs are conducted on populations with similar balances of cases which show a presence or absence of relaxation of egg output constraints, then they will be comparable despite the % FECR values underestimating actual drug efficacy. However, if the degree of pre-treatment constraint is different between two populations, then the potential exists for % FECR values to falsely indicate different drug efficacy between the two populations.

Removal of the most heavily infected human cases whose worm populations are significantly constrained by density dependent effects from a FECRT study would seem desirable in order to reduce the potential distorting effects that relaxation of the constraints in these cases may have on the FECRT data. Anderson and Schad [20], working with mixed infections of *N. americanus* and *A. duodenale*, showed that, although density dependent fecundity resulted in a poor relationship between epg and worm burden, it was possible to discriminate between people with very low and very high infections on the basis of epg. This indicates that omission of high burden cases with potential to distort FECRTs would be possible. However, our analysis of the Hill [18] *N. americanus* data set in this regard shows that such selection of suitable candidates for a FECRT may not always be so clear. For the Hill [18] dataset, omission of the highest epg cases would not result in preferential removal of the highest worm burden cases (from Figure 3A). It is apparent that the increase in fecundity seen at lower worm burdens is of such a magnitude that the total egg output by these less dense populations (as measured by epg) can greatly exceed that of populations containing significantly higher numbers of worms. However, for the Hill [18] data set, as the epg cut-off for omission from a FECRT was further reduced, the removal of most heavy infections was evident (Figures 3B, 3C). A negative outcome is that many lighter infection cases were also removed. The cost, in terms of necessitating the omission of many apparently ‘unconstrained’ populations, may be outweighed by the benefit of removing the potential distorting effects of the heavy infection cases. Decisions on omission of cases for FECRTs may depend on local infection levels, and availability of suitable numbers of people to satisfy the statistical requirements of the test. Further study may identify epg cut-off values that could be applied widely to FECRTs.

The FECRT is used in livestock industries as a measure of drug resistance. The question arises as to whether the density dependent effects we have described will have a significant potential to distort FECRTs with the human parasites given the acceptance of the utility of the test for livestock. While reports on some livestock species have shown an absence of density dependent effects [10],[11], other livestock species show strong density dependence (for example, *Teladorsagia circumcincta* [34]). A difference in the application of FECRTs to livestock and humans, which may influence the impact of density dependency, is the efficacy levels of anthelmintics in the two systems. In livestock, the expected drug efficacy for susceptible parasites is greater than 99%. Resistance is suspected if this value drops below 95% (and if the lower 95% confidence interval is below 90%) [35]. Hence, very little of a susceptible worm population within each host is expected to survive the drug treatment, leaving little scope for egg output in the remaining worms to be significantly amplified by relaxation of density dependent constraints. Hence, a susceptible worm population is clearly indicated by the test. The effect of a relaxation of density dependent fecundity constraints in amplifying egg counts from worms remaining after drug treatment may only be expected if a significant proportion of the population is drug resistant. In humans, however, the situation is quite different. Efficacies for some human anthelmintics would never have originally met the criteria required by the livestock FECRT as indicating susceptibility, suggesting the use of suboptimal dosing regimes in some cases. While egg reduction rates for *A. lumbricoides* after treatment with albendazole or mebendazole are generally close to 100%, the values for hookworms and *Trichuris* are much lower [36],[37]. For example, in the analysis of a number of studies by Keiser and Utzinger [37], hookworm egg reduction rates in response to albendazole varied form 64.2% to 100%, with an overall cure rate of 78.4%. Hence, with significant proportions of susceptible worms expected to survive current treatment regimes, the capacity for the relaxation of fecundity constraints in drug susceptible worms to distort efficacy measurements may be significantly greater than would be expected for the livestock parasite species.

Given the potential for distortion of FECRT data by density dependent fecundity, it may be difficult to identify apparent drug efficacy changes (that is, the development of drug resistance) amongst a background of dynamic female worm reproductive biology. The impact of worm biology on the utility of the FECRT as a resistance detection tool highlights the need to remove this influence by developing methods which examine either direct drug effects on isolated worms with *in vitro* phenotypic assays, or changes in worm genotypes, as drug resistance monitoring tools.
